# Pickering Emulsion of Oleoresin from *Dipterocarpus alatus* Roxb. ex G. Don and Its Antiproliferation in Colon (HCT116) and Liver (HepG2) Cancer Cells

**DOI:** 10.3390/molecules29112695

**Published:** 2024-06-06

**Authors:** Piman Pocasap, Kawintra Tamprasit, Thanyathanya Rungsri, Karnchanok Kaimuangpak, Tarapong Srisongkram, Somporn Katekaew, Khanita Kamwilaisak, Ploenthip Puthongking, Natthida Weerapreeyakul

**Affiliations:** 1 Department of Pharmacology, Faculty of Medicine, Khon Kaen University, Khon Kaen 40002, Thailand; pimapo@kku.ac.th; 2 Graduate School in the Program of Research and Development in Pharmaceuticals, Faculty of Pharmaceutical Sciences, Khon Kaen University, Khon Kaen 40002, Thailand; ta.kawintra@kkumail.com (K.T.); karkai@kkumail.com (K.K.); 3 Research Institute for Human High Performance and Health Promotion, Khon Kaen University, Khon Kaen 40002, Thailand; tarasri@kku.ac.th; 4 Faculty of Pharmaceutical Sciences in the Program of Doctor of Pharmacy, Khon Kaen University, Khon Kaen 40002, Thailand; thanyathanya_r@kkumail.com; 5 Division of Pharmaceutical Chemistry, Faculty of Pharmaceutical Sciences, Khon Kaen University, Khon Kaen 40002, Thailand; pploenthip@kku.ac.th; 6 Department of Biochemistry, Faculty of Sciences, Khon Kaen University, Khon Kaen 40002, Thailand; somkat@kku.ac.th; 7 Department of Chemical Engineering, Faculty of Engineering, Khon Kaen University, Khon Kaen 40002, Thailand; khanita@kku.ac.th

**Keywords:** Pickering emulsion, oil-in-water, *Dipterocarpus alatus*, anticancer activity, cell-based assay

## Abstract

Oleoresin of *Dipterocarpus alatus* Roxb. ex G. Don (DA) has been traditionally used for local medicinal applications. Several in vitro studies have indicated its pharmacological potential. However, the low water solubility hinders its use and development for pharmaceutical purposes. The study aimed to (1) formulate oil-in-water (*o*/*w*) Pickering emulsions of DA oleoresin and (2) demonstrate its activities in cancer cells. The Pickering emulsions were formulated using biocompatible carboxylated cellulose nanocrystal (cCNC) as an emulsifier. The optimized emulsion comprised 3% (F1) and 4% (*v*/*v*) (F2) of oleoresin in 1% cCNC and 0.1 M NaCl, which possessed homogeneity and physical stability compared with other formulations with uniform droplet size and low viscosity. The constituent analysis indicated the presence of the biomarker dipterocarpol in both F1 and F2. The pharmacological effects of the two emulsions were demonstrated in vitro against two cancer cell lines, HepG2 and HCT116. Both F1 and F2 suppressed cancer cell viability. The treated cells underwent apoptosis, as demonstrated by distinct nuclear morphological changes in DAPI-stained cells and Annexin V/PI-stained cells detected by flow cytometry. Our study highlights the prospect of Pickering emulsions for oleoresin, emphasizing enhanced stability and potential pharmacological advantages.

## 1. Introduction

*Dipterocarpus alatus* Roxb. ex G. Don (DA) is a sizable tree species exclusive to the Southeast Asian mainland, valued for timber and resin extraction [1]. Ethnobotanically, it has been historically utilized to address various ailments, such as genitourinary, skin-related issues, and inflammatory conditions [2]. The medicinal attributes of DA have been extensively documented, predominantly stemming from its oleoresin—the most frequently utilized derivative of the plant. Previous studies have highlighted the in vitro pharmacological potential of DA oleoresin, showcasing its cytotoxicity against diverse cancer cell lines, including lung (SK-LU-1), skin (SK-MEL-2), and cervical (U937) cells, while demonstrating apoptosis induction in leukemic cells (Jurkat) [3,4]. Additionally, oleoresin exhibits notable antioxidant and antidepressant properties [4,5]. Among its constituents, sesquiterpenes emerge as the primary chemical classified within the oleoresin, with dipterocarpol identified as a key biologically active component [3,6]. Despite its promising attributes, the utility of DA oleoresin is hampered by its inherent limitation: immiscibility with water, which restricts its application scope.

Oleoresin is a natural semisolid mixture containing hydrophobic substances and resin. The hydrophobic properties render oleoresin water miscibility and its use in aqueous-related applications [7]. Several approaches have been reported to enhance the water miscibility of substances or mixtures for clinical purposes. One of the distinct pharmaceutical approaches—known to enhance the properties without altering chemicals or compositions of its contents—is emulsification, the process of dispersing two immiscible liquids by forming an emulsion [8]. Emulsion increases its content water miscibility by encapsulating the hydrophobics within tiny droplets (oil phase) dispersed throughout the water phase. The process requires the use of an emulsifier, amphiphilic molecules, to stabilize the emulsion by forming a stable interface between two immiscible liquids [9]. Conventional emulsion typically uses surfactant as an emulsifier. As an alternative, Pickering emulsion uses solid particles as an emulsifier and potentially yields enhanced stability due to the nature of solid particles. We previously reported the formulation of Pickering emulsions using biocompatible carboxylated cellulose nanocrystal (cCNC) as an emulsifier [10,11]. The emulsions displayed enhanced physical stability and aqueous compatibility while still retaining the biological activity of its hydrophobic contents—implying the application of Pickering emulsion for modifying properties of hydrophobic mixtures, such as oleoresin.

The objectives of the study were, therefore, to formulate a Pickering emulsion containing DA oleoresin using biocompatible cCNC as an emulsifier and demonstrate the emulsion’s activity in an aqueous environment using cell-based assays. The emulsion composition, including oleoresin, salt (NaCl), and emulsifier, was optimized based on the emulsion’s homogeneity and physical stability. The stability of formulations was also investigated in various storage conditions. The selected emulsions were then characterized for physical properties and content to confirm the formation of Pickering emulsion. The activity of the selected emulsions on cell viability and apoptosis induction was further demonstrated in cancer cell lines HepG2 and HCT116. The outcome of the study not only presents the formulation of Pickering emulsion for hydrophobic oleoresin but also implies Pickering emulsion as an attractive choice for formulating and delivering hydrophobic substances.

## 2. Results

### 2.1. Emulsion Formulation

**Figure 1:**
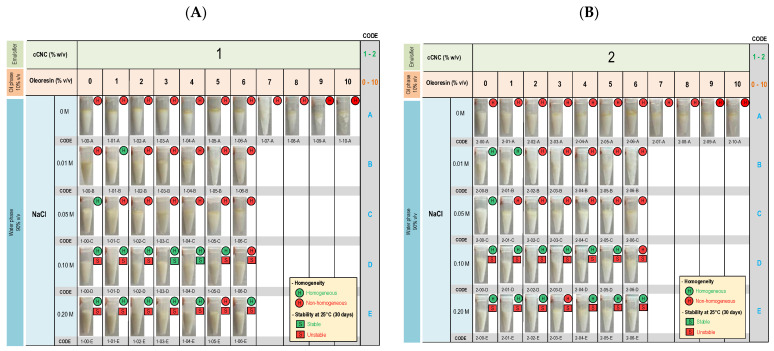
Physical appearance of DA oleoresin Pickering emulsion with 1% cCNC (**A**) and 2% cCNC (**B**) as emulsifier. The emulsions were formulated by varying the oleoresin ratio in the oil phase (10% total) and NaCl in the water phase (90%). Following preparation and storage (25 °C for 30 days), the physical stability of the emulsions was assessed through visual observation.

Pickering emulsions were formulated with the ratio of the water-to-oil phase of 9:1 using cCNC as emulsifiers. The oil phase consists of both plant oil and oleoresin. First, to determine the optimum ratio between plant oil and oleoresin, the formulation coded 1-00-A to 1-10-A and 2-00-A to 2-10-A were formulated (without the addition of NaCl in the water phase). The maximum concentration of oleoresin was 6% because the higher oleoresin ratio resulted in emulsion flocculation and coalescence (Figure 1). In addition, a yellowish oil on the emulsion surfaces was observed, indicating that cCNC might not effectively trap the oleoresin within the emulsion particles.

The impact of salt (NaCl) was then explored in formulations containing less than 6% oleoresin (formulations 1-00-B to 2-06-E) with NaCl concentrations ranging from 0.01 to 0.2 M. Incorporating 0.1 and 0.2 M NaCl mostly resulted in compatible systems, while formulations with NaCl concentrations below 0.1 M showed physical instability, such as flocculation and coalescence. The stability of formulation with 0.1 M and 0.2 M NaCl was investigated in a storage condition. Stability tests at 25 °C for 30 days demonstrated that formulations 1-03-D and 1-04-D remained physically stable under the condition (Figure 1 and Supplementary Materials: Table S2).

**Figure 2:**
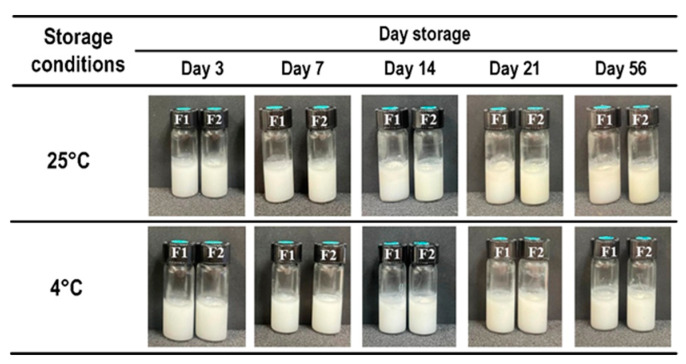
The physical changes in Pickering emulsions F1 and F2 during storage at 25 °C and 4 °C for 56 days.

Extended stability assessments over 56 days revealed that both formulations 1-03-D and 1-04-D maintained physical stability without phase separation at both 25 °C and 4 °C (Figure 2). As a result of their sustained stability, formulations 1-03-D (F1) and 1-04-D (F2) were selected for further analysis.

### 2.2. Homogeneity of Formulations

**Figure 3:**
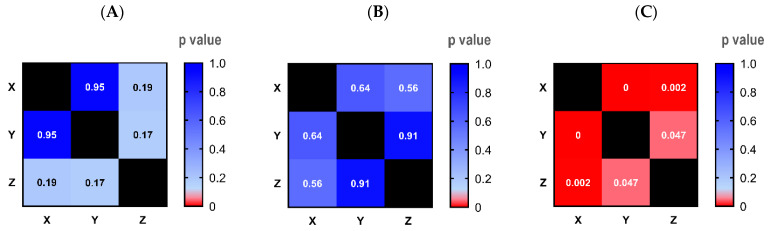
Homogeneity test by image analysis. Homogeneity was assessed via image analysis, comparing the intensity of areas X, Y, and Z among formulations F1 (**A**), F2 (**B**), and formulation 1-01-A (used as a positive control) (**C**). A higher *p*-value indicates increased uniformity among these areas.

The homogeneity of Pickering emulsion F1 and F2 was additionally validated using the image analytical method. Figure 3 displays the *p*-value from statistical analysis of images among spots (A, B, and C) on each formulation. In contrast with formulation coded 1-01-A (as a positive control), there is no difference among spots in emulsion F1 and F2. These data strongly suggest the homogeneity of these formulations, as demonstrated by the image analysis.

### 2.3. Formulation’s Characteristics

**Figure 4:**
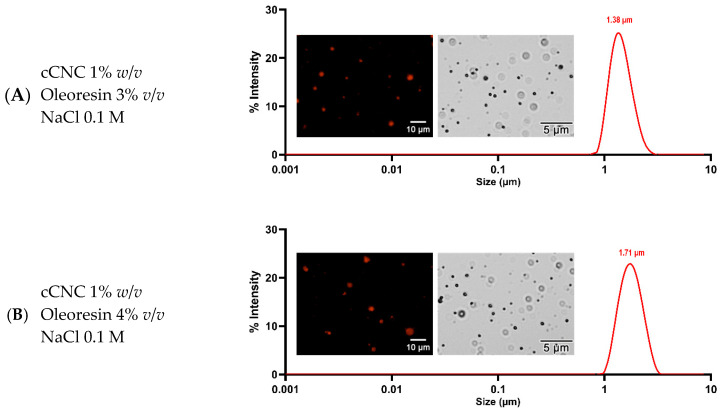
Fluorescence microscopic image of Pickering emulsions F1 (**A**) and F2 (**B**) stained with oil red-O dye and their size distribution.

The properties of Pickering emulsions F1 and F2 were assessed, including particle size, polydispersity index (PDI), and macro-rheological parameters. Both formulations display similar emulsion characteristics (Table 1 and Figure 4). The average particle size of both formulations is approximately 1 μm with a monomodal size distribution. The data are in accordance with the obtained PDI value (0.2–0.3 mV), which indicates the particle size distribution—ranging from 0 (perfect homogeneity) to 1 (high heterogeneity)—suggesting the high homogeneity of the droplet size of both formulations. Macro-rheological parameters such as shear stress and rate were measured to determine the viscosity of the emulsions. Both F1 and F2 exhibited low viscosity of around 9.7 cP, along with moderate shear stress (~0.83 dyne/cm^2^) and shear rate (8.5 s^−1^), indicating their less viscous and more fluid-like behavior. To evaluate the oil-phase composition within the particles (comprising oleoresin and plant oil), Red-O, a biological dye for lipid staining, was utilized. The images in Figure 4 displayed the presence of oil components encapsulated within the particles, confirming the formation of o/w emulsions.

### 2.4. Dipterocarpol Contents

The standard biomarker for DA, dipterocarpol, was analyzed using HPLC. In our prior study, dipterocarpol within DA oleoresin was identified through H1- and C13-NMR spectroscopy and quantified using HPLC [3]. Correspondingly, in this research, dipterocarpol was detected in the oleoresin at a concentration of 95.31 mg/mL. Furthermore, dipterocarpol was identified in Pickering emulsions F1 (5.72 mg/mL) and F2 (7.62 mg/mL) (Supplement Figure S1).

### 2.5. Effects of Pickering Emulsions of Oleoresin on Cancer Cell Viability

**Figure 5:**
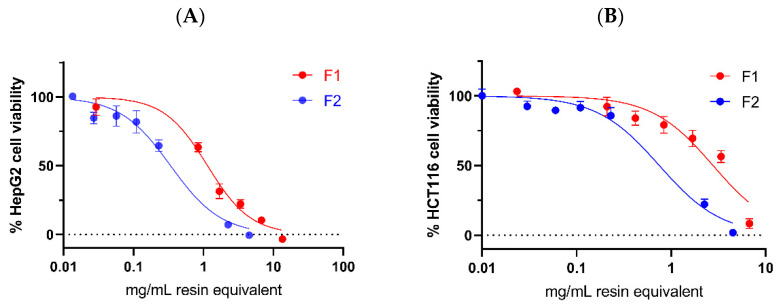
Cell viability in the HepG2 (**A**) and HCT116 (**B**) cancer cell lines following treatment with Pickering emulsions F1 and F2 for 24 h. The values represent the mean ± SD of triplicate measurements.

The impact of Pickering emulsions F1 and F2 on cell viability in two cancer cell lines, hepatocellular carcinoma HepG2 and colorectal carcinoma HCT116, was evaluated. Both F1 and F2 exhibited a dose-dependent reduction in cancer cell viability against both cell lines, as depicted in Figure 5. Table 2 presents the IC_50_ values of F1, F2, and DA oleoresin, along with standard compounds—dipterocarpol and cisplatin—against HepG2 and HCT116. Notably, the Pickering emulsion without oleoresin showed no observable effect.

### 2.6. Effects of Pickering Emulsions of Oleoresin on Nuclear Morphology Change

**Figure 6:**
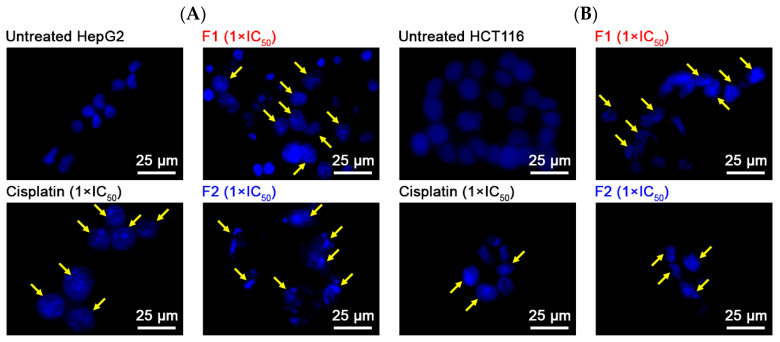
Fluorescent microscopy was conducted on HepG2 (**A**) and HCT116 (**B**) cells stained with DAPI after being exposed to Pickering emulsions F1, F2, and cisplatin (positive control) at their respective IC_50_ concentrations for 24 h. The 1 × IC_50_ values for F1 and F2 against HepG2 cells were 1.25 and 0.92 mg/mL, respectively. Against HCT116 cells, the 1 × IC_50_ values for F1 and F2 were 3.45 and 1.77 mg/mL, respectively.

The nuclear morphology of the two cancer cell lines was further examined following treatment with Pickering emulsions F1 and F2. The outcomes demonstrated that untreated control cells (HepG2 and HCT116) exhibited consistent blue fluorescence, indicating uniform chromatin distribution within the nucleus. This suggests an absence of DNA fragmentation and cell death. Conversely, treatment with cisplatin (a positive control), F1, and F2 (at 1 × IC_50_) led to notable alterations in nuclear morphology in both HepG2 and HCT116 cells. These alterations included enlarged nuclei and nuclear fragmentation, indicative of the activation of apoptotic processes (Figure 6).

### 2.7. Effects of Pickering Emulsions of Oleoresin on Apoptosis Induction

**Figure 7:**
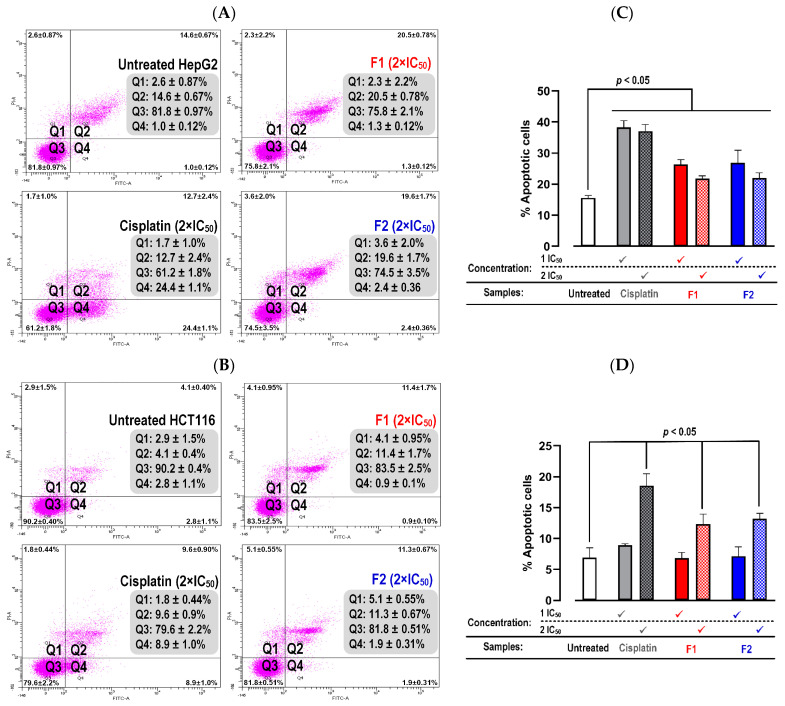
Apoptotic cell death induced by Pickering emulsions F1 and F2. Dot plots illustrate the mode of cell death in HepG2 (**A**) and HCT116 (**B**) after 24 h of F1 and F2 treatment. Quadrants 1 (Q1) to 4 (Q4) indicate necrosis, late apoptosis, viable cells, and early apoptosis, respectively. Total apoptotic cell death in HepG2 (**C**) and HCT116 (**D**) after F1 and F2 treatment for 24 h was quantified. Values are presented as the mean ± SD of triplicate measurements, and significance was determined at a *p*-value < 0.05. The 2 × IC_50_ values for F1 and F2 against HepG2 cells were 2.50 and 1.84 mg/mL, respectively. Against HCT116 cells, the 2 × IC_50_ values for F1 and F2 were 6.90 and 3.54 mg/mL, respectively.

The apoptosis induction activity of Pickering emulsions F1 and F2 (at concentrations of 1 × IC_50_ and 2 × IC_50_) was determined in HepG2 and HCT116. Cisplatin, as well as F1 and F2, increased HepG2 and HCT116 apoptotic cell death, as indicated by enhanced cell populations in Q2 (early-stage apoptosis) and Q3 (late-stage apoptosis) in dot plots (Figure 7A,B. Total apoptosis (sum of early- and late-stage apoptosis) was compared with the untreated control and represented as a bar plot (Figure 7C,D). Our data indicates that both F1 and F2 increased total apoptotic cell death in HepG2 and HCT116. The pattern of dose dependence was also observed in HCT116-treated cells.

## 3. Discussion

Several medicinal properties linked to DA oleoresin have been extensively studied in vitro. Our initial findings revealed an issue with the water immiscibility of DA oleoresin, complicating its extraction and dilution processes (Supplement Table S1). This immiscibility poses considerable challenges, hindering comprehensive exploration of the oleoresin using advanced experimental models and restricting its practical applications. Therefore, in this study, we introduce Pickering emulsions as a novel formulation method aimed at enhancing the compatibility of resinous substances in aqueous environments, offering an alternative for biological studies.

Carboxylate-cellulose nanocrystal (cCNC) was prepared and used as emulsifiers (1% and 2% *w*/*v*) for the formulation of o/w emulsion, considering its preferable pharmaceutical properties and biocompatibility [11,12]. Maintaining a fixed ratio of 9 to 1 for the water-to-oil phase, our initial focus was on optimizing the composition ratios within the oil phase, encompassing plant oil and oleoresin. Our investigations indicated that the maximum permissible concentration of oleoresin stood at 6%. Exceeding this concentration led to flocculation and coalescence. Notably, despite the concentration limit, we observed stratification within the oil phase, signifying that the emulsifier alone was insufficient in stabilizing all the oil particles within the emulsion droplets. The addition of salt was previously reported to enhance the stability of Pickering emulsion by increasing electrostatic repulsions between particles and strengthening interfacial layers [13]. Accordingly, we varied the concentration of NaCl in the water phase. NaCl concentrations of 0.1 M and 0.2 M resulted in homogeneous systems, suggesting that the NaCl salt bolstered the cCNC’s ability to encapsulate the oil particles within the emulsion droplets. Consequently, the physical stability of the formulations was determined under storage conditions, specifically 25 °C and 4 °C for 21 days. We found that two formulations—F1 and F2—were stable under the storage conditions. The extended storage conditions for 56 days were then performed on the two formulations. F1 and F2 were both stable under the extended conditions. And considering the formulation homogeneity and stability, F1 and F2 were selected for further analysis: characterization.

The homogeneity of Pickering emulsion formulations F1 and F2 was confirmed visually and through image analysis. Red-O staining revealed the encapsulation of oil components within the droplets, while HPLC analysis detected dipterocarpol, the oleoresin biomarker of DA in both F1 and F2. These results strongly suggest the successful formation of o/w emulsions of oleoresin. Physical characterization revealed that both F1 and F2 exhibited droplet sizes around 1 μm with a monomodal distribution, classifying them as macroemulsions with uniform droplet size [14]. The macroemulsion offers good kinetic stability (i.e., stability over time) since the effect of gravitational force, as well as Brownian motions, are relatively weak on a large droplet [15,16]. The size uniformity also has less tendency to Ostwald ripening (redepositing of smaller onto larger droplets) since there is less surface area and energy between droplets, resulting in better resistance against coalescence [17]. Notably, in exchange for enhanced stability, due to the lower surface area to volume ratio of macroemulsion—in contrast with nano/microemulsion—the droplet content is supposed to be released lower [18]. The rheological parameters indicate that F1 and F2 possess fluid-like behavior that is suitable for liquid food and pharmaceutical products. The ease of handling is also an advantage of the lower viscous emulsions, which match our preference for sample handling, an in vitro study to demonstrate the efficacy of the Pickering emulsions.

The use of solid particles as emulsifiers in Pickering emulsions offers significant advantages in specific areas. Cellulose, acting as an emulsifying agent, forms a mechanical protective barrier at the interface, enhancing emulsion stability by preventing particle coalescence and lipid peroxidation [19,20]. However, this barrier might affect the release of contents, potentially impeding the activity of emulsion ingredients, either by becoming entangled within the emulsifier’s polymer chains or staying adsorbed on emulsion surfaces [21]. To address this concern, we conducted in vitro assessments of oleoresin Pickering emulsions using cell-based assays. Cell viability assays revealed that F1 and F2 were compatible with aqueous systems and could reduce the viability of both HepG2 and HCT116 cancer cell lines, while Pickering emulsion lacking oleoresin showed no cytotoxic effects. These findings suggest that the impact on cell viability stemmed from the oleoresin, not other components in the Pickering emulsion. Further investigation using DAPI staining for nuclear morphology indicated a qualitative link between F1 and F2 treatments and potential cancer cell death through apoptosis. Quantitative assessment through flow cytometry analysis using Annexin V/PI staining confirmed increased apoptotic cell death in both HepG2 and HCT116 cell lines after exposure to F1 and F2. This collective evidence supports our demonstration that Pickering emulsions F1 and F2 could release droplet contents, exerting a pharmacological action in the process.

The pharmacological activities of DA oleoresin have been previously reported. The data in this study is in accordance with our previous report confirming the activity of oleoresin on apoptosis induction. Interestingly, the IC_50_ of oleoresin (~0.1 mg/mL) in cell viability assay—dissolving in aqueous medium using DMSO as a dispersing agent—from our previous report [3] displays ten times lower in contrast with the Pickering emulsion F1 and F2 (~1 mg/mL) against HepG2. The data aligns with our current findings, indicating that oleoresin without Pickering emulsion exhibits a lower IC_50_. Several reports indicate the sustained release property of Pickering emulsion using cellulose nanocrystal as an emulsifier [22,23]. Encapsulated coumarin and curcumin in cellulose nanocrystal display a sustained release profile with in vitro anticancer activity [24]. The sustained release behavior of citrus oil from Pickering emulsion was proportional to the concentration of cellulose nanocrystals, forming an interfacial barrier and delaying component diffusion [25]. Therefore, in this study, we surmise that cellulose nanocrystals sustained the release of oleoresin, which resulted in a higher IC_50_ due to the cellulose’s mechanical barrier. Nevertheless, to confirm the statement, a content-releasing profile and an in vitro study with a longer duration should be performed.

In terms of safety, our findings indicate that Pickering emulsion without oleoresin is safe, consistent with previous studies demonstrating the low toxicity and excellent biocompatibility of cellulose-based Pickering emulsions [26,27]. However, the safety of the oleoresin itself remains uncertain. Our previous reports show that the selective index (SI), comparing the normal cell line Vero against several cancer cell lines (HCT116, SK-LU-1, SK-MEL-2, SiHa, and U937), averages around 0.8 [4]. An SI above 2 is generally considered safe [28], but our values indicate a narrower therapeutic window, raising concerns about potential adverse effects on non-cancerous cells. The selective cytotoxicity observed may result from specific interactions between the oleoresin’s bioactive compounds and cellular components, which vary among cell types. While DA oleoresin exhibits promising anticancer activity, its cytotoxicity towards normal cells presents a significant challenge for systemic treatments. One promising approach to enhancing safety involves using stimulus-responsive drug release systems. These advanced systems respond to triggers such as pH, temperature, light, or enzymes [29], which differ between cancerous and normal tissues, providing precise control over drug delivery. For instance, cellulose-based Pickering emulsions can exploit the acidic microenvironment of tumors for selective chemical release [30]. Temperature-controlled release has also been reported, allowing targeted delivery in response to localized hyperthermia [31]. These strategies improve the therapeutic index by minimizing exposure to healthy cells and reducing systemic toxicity. Nevertheless, while Pickering emulsions without oleoresin show a favorable safety profile, the oleoresin’s cytotoxicity towards normal cells highlights the need for further refinement of formulations and delivery methods to ensure safe and effective therapeutic use.

## 4. Materials and Methods

### 4.1. Chemicals

Carboxylated cellulose nanocrystals (cCNCs) were derived from eucalyptus pulp cellulose and underwent characterization following procedures outlined in a prior study [11]. Briefly, one gram of sieved pulp was mixed with 20 mL of 50% sulfuric acid solution, sonicated for 15 min, and hydrolyzed at 60 °C for 60 min. After centrifugation at 9000 rpm, the solid phase (CNC) was neutralized with distilled water and dried at 60 °C for 24 h. Analytical reagents methanol (V.S. Chem House, Bangkok, Thailand), 37% hydrochloric acid (HCl) (RCI Labscan, Bangkok, Thailand), sodium chloride (ACL Lab Scan Co. Ltd., Bangkok, Thailand), and HPLC-grade acetonitrile and trifluoroacetic acid (V.S. Chem House) were used without modification. Additionally, materials including Dulbecco’s modified Eagle’s medium with high glucose (DMEM), penicillin/streptomycin solution (100×), fetal bovine serum (FBS), and 0.25% trypsin-EDTA (1×) were procured from GIBCO**^®^** (Barcelona, Spain). Neutral red (NR) was purchased from Sigma Chemical Co. (St. Louis, MO, USA), and the standard anticancer drug cisplatin was obtained from Boryung (Ansan, Korea).

### 4.2. Plant Material

The oleoresin was collected in 2021 from Wong Tawee Farm in Chon Buri province, Thailand, and originated from 27 to 30-year-old trees standing at heights of 30 to 50 m. Its preparation followed the methods detailed in a previous report [4]. To maintain the oleoresin’s compositional integrity throughout the study, the oleoresin was stored at −20 °C in the absence of light.

### 4.3. Formulation of Pickering Emulsion

Pickering Emulsion from DA oleoresin was prepared according to a previous report [11] with minor modifications. Briefly, the oil-in-water solution (1:9 *v/v* ration) was mixed with cCNC and sonicated by an ultrasonic bath (Model Lab855, Sl Analytics, Washington, DC, USA). Pickering emulsion of oleoresin (o/w) emulsion was then optimized for the concentrations of cCNC, NaCl, and oil phase (plant oil and oleoresin). The optimal formulations were determined based on homogeneity and physical stability. The emulsion compositions formulated in this study are displayed in Table 3.

### 4.4. Homogeneity Test by Image Analysis

Homogeneity analysis utilized the ImageJ software (U.S. NIH, Bethesda, MD, USA). In brief, images of individual samples were captured, focusing on three designated areas (A, B, and C). The intensity of 90 randomly selected points within each area underwent measurement, and statistical significance was assessed using one-way ANOVA. The emulsion’s homogeneity was confirmed when no statistical differences were observed across all selected areas.

### 4.5. Particle Size, Polydispersity Index, Macro-Rheological Measurements

The microscopic image of Pickering emulsion droplets was examined through optical microscopy. The emulsion (30 µL) was deposited onto a glass slide, and an inverted microscope (AE2000, MoticEurope, Barcelona, Spain) was used to capture images of the emulsion droplets. The droplet size and polydispersity index (PDI) of the Pickering emulsion were then assessed employing dynamic light scattering. For the analysis, emulsions were diluted 100× and introduced into disposable cuvettes (Malvern Panalytical, UK). The particle size was determined via noninvasive backscatter (NIBS) utilizing a Zetasizer Nano ZS (Malvern Panalytical, UK) in the manual duration mode at 25 °C. Data collection and analysis were performed using Zetasizer software (version 7.13, Malvern Panalytical, UK). Rheological measurements were conducted using a Brookfield viscometer (Brookfield model DV-II and viscometer, USA). A volume of 10 mL of Pickering emulsion was analyzed for shear rate and shear stress at a temperature of 25 °C and a speed of 25 rpm. All measurements were executed in triplicate.

### 4.6. Dipterocapol Contents

The Pickering emulsion and DA oleoresin were extracted by methanol (ratio 1:1), followed by a 10-min sonication and centrifugation at 2800× *g* for 10 min to gather the supernatant. This extraction process was repeated thrice using the same pellet. Subsequently, solvent removal was carried out utilizing the SpeedVac DDA concentrator Model SPD140DDA-230 (ThermoFisher Scientific, Newington, NH, USA). The HPLC analysis of dipterocarpol content was conducted using an LC–2030C3D quaternary pump (Shimadzu, Kyoto, Japan) paired with a diode array detector (DAD), following previously reported methods [3]. Briefly, samples were injected into a HiQ sil C18W column (4.6 mm × 250 mm, 5 μm) (KYA Technologies Corporation, Tokyo, Japan). The isocratic mobile phase consisted of 0.01% trifluoroacetic acid in ultrapure water (solvent A) and acetonitrile (solvent B) at a fixed ratio of 5:95% (solvent A: solvent B). The column temperature was maintained at 25 °C, with a flow rate of 1 mL/min, and the detector wavelength was set at 210 nm.

### 4.7. Cell Culture

Hepatocellular carcinoma (HepG2) and human colorectal carcinoma (HCT116) were cultured in DMEM. The media were supplemented with 10% fetal bovine serum (FBS), 100 units/mL penicillin, and 100 µg/mL streptomycin. The cell lines were incubated at 37 °C with 95% air and 5% CO_2_ and reached approximately 80% confluent prior to use.

### 4.8. Cell Viability by Neutral Red Assay

Cell viability assessments were conducted using the NR assay, as previously described [4]. HepG2 cells (4.5 × 10^5^ cells/mL) and HCT116 cells (4 × 10^5^ cells/mL) were seeded into 96-well plates. Subsequently, these cells were exposed to varying concentrations of samples for 24 h. After treatment, the cells underwent washing with PBS and were then subjected to incubation with neutral red (final concentration: 50 µg/mL) for 2 h at 37 °C. Following this incubation, the supernatant was removed, and the neutral red incorporated within the cells was lysed using 0.33% HCl in isopropanol. The absorbance was measured using a microplate reader at 537 nm (reference wavelength: 650 nm). The IC_50_ was calculated from a plot of % cell viability and sample concentrations.

### 4.9. Nuclei Morphological Alteration by DAPI Staining

Nuclear morphology assessment was conducted using DAPI staining, following a previous report [32]. HepG2 and HCT116 cells (1 × 10^4^ cells/mL) were cultured in 24-well plates and exposed to samples for 24 h. Subsequently, the culture media were discarded, and cell washing was performed using PBS. The cells were then fixed using cold MeOH for 30 min. Methanol was then removed, and 0.3 µg/mL DAPI in PBS was added and incubated for at least 1 h. The excess dye was removed, and PBS:glycerin (1:1) was added prior to observing under an inverted fluorescent microscope at 80× magnification.

### 4.10. Mode of Cancer Cell Death Using Flow Cytometry

The mode of cancer cell death was determined using Annexin V FITC/Propidium iodide (PI) staining and detected by flow cytometry, as per the manufacturer’s instruction BioLegend (San Diego, CA, USA). Briefly, the treated cells were harvested and re-suspended in a binding buffer (1×). Annexin V and PI were then added, followed by a 15-min incubation at room temperature in darkness. The stained cells were analyzed using a fluorescent-activated cell sorter (FACS) analyzer (BD FACSCanto II, Franklin Lakes, NJ, USA).

### 4.11. Statistical Analysis

The data were presented as mean ± SD. Variations among treatments were assessed through one-way ANOVA followed by a Tukey’s multiple comparison post hoc test, conducted using SPSS 28.0 for Windows**^®^** (SPSS Inc., Chicago, IL, USA). Differences in *p*-values below 0.05 were considered significant.

## 5. Conclusions

This study demonstrated the use of Pickering emulsion with cCNC as an emulsifier to enhance the compatibility of DA oleoresin for biological studies. Emulsions F1 (3% oleoresin) and F2 (4% oleoresin) were selected for their homogeneity and physical stability. Analysis confirmed the presence of dipterocarpol, a phytochemical biomarker of DA, in both formulations. Characterization showed that F1 and F2 formed o/w emulsions with uniform droplet size and low viscosity. Cell-based assays indicated that both F1 and F2 reduced cancer cell viability in HepG2 and HCT116 cell lines and induced apoptosis, as confirmed by nuclear morphological changes and flow cytometry. The study highlights the potential of Pickering emulsions with cellulose nanocrystals as effective carriers for oleoresin, offering improved stability and pharmacological benefits. Further research on content release profiles and extended pharmacological evaluations is recommended.

## Figures and Tables

**Table 1:** The average drop diameter, polydispersity, and macro-rheological measurements of the prepared Pickering emulsions F1 and F2.

Formulation	Particle Size (μm)	PDI	Viscosity (cP)	Sheer Stress (dyne/cm^2^)	Sheer Rate (s^−1^)
F1	1.38 ± 0.079	0.30 ± 0.050	9.6 ± 1.22	0.82 ± 0.03	8.5 ± 0.15
F2	1.71 ± 0.14	0.23 ± 0.026	9.8 ± 1.4	0.84 ± 0.01	8.5 ± 0.15

Data are expressed as the mean and SD of triplicate measurements.

**Table 2:** Half maximal inhibitory concentration (IC_50_) of F1 and F2 on cancer cell viability.

Samples	IC_50_ (mg/mL)	
	HepG2	HCT116
Cisplatin	0.0268 ± 0.0015	0.0948 ± 0.0032
DA oleoresin	0.247 ± 0.014	0.340 ± 0.021
Pickering emulsion without oleoresin	* Inactive	* Inactive
F1 (mg/mL resin equiv.)	1.25 ± 0.27	3.45 ± 0.26
F2 (mg/mL resin equiv.)	0.92 ± 0.05	1.77 ± 0.07

* Inactive is indicated as having IC_50_ greater than 100 mg/mL (the maximum concentration used).

**Table 3:** Pickering emulsion compositions.

Formulation	cCNC (%*w*/*v*)	Oleoresin (%*v*/*v*)	NaCl (M)	Formulation	cCNC (%*w*/*v*)	Oleoresin (%*v*/*v*)	NaCl (M)
1-00-A	1	0	0	2-00-A	2	0	0
1-01-A	1	1	0	2-01-A	2	1	0
1-02-A	1	2	0	2-02-A	2	2	0
1-03-A	1	3	0	2-03-A	2	3	0
1-04-A	1	4	0	2-04-A	2	4	0
1-05-A	1	5	0	2-05-A	2	5	0
1-06-A	1	6	0	2-06-A	2	6	0
1-07-A	1	7	0	2-07-A	2	7	0
1-08-A	1	8	0	2-08-A	2	8	0
1-09-A	1	9	0	2-09-A	2	9	0
1-10-A	1	10	0	2-10-A	2	10	0
1-00-B	1	0	0.01	2-00-B	2	0	0.01
1-01-B	1	1	0.01	2-01-B	2	1	0.01
1-02-B	1	2	0.01	2-02-B	2	2	0.01
1-03-B	1	3	0.01	2-03-B	2	3	0.01
1-04-B	1	4	0.01	2-04-B	2	4	0.01
1-05-B	1	5	0.01	2-05-B	2	5	0.01
1-06-B	1	6	0.01	2-06-B	2	6	0.01
1-00-C	1	0	0.05	2-00-C	2	0	0.05
1-01-C	1	1	0.05	2-01-C	2	1	0.05
1-02-C	1	2	0.05	2-02-C	2	2	0.05
1-03-C	1	3	0.05	2-03-C	2	3	0.05
1-04-C	1	4	0.05	2-04-C	2	4	0.05
1-05-C	1	5	0.05	2-05-C	2	5	0.05
1-06-C	1	6	0.05	2-06-C	2	6	0.05
1-00-D	1	0	0.1	2-00-D	2	0	0.1
1-01-D	1	1	0.1	2-01-D	2	1	0.1
1-02-D	1	2	0.1	2-02-D	2	2	0.1
1-03-D *	1	3	0.1	2-03-D	2	3	0.1
1-04-D **	1	4	0.1	2-04-D	2	4	0.1
1-05-D	1	5	0.1	2-05-D	2	5	0.1
1-06-D	1	6	0.1	2-06-D	2	6	0.1
1-00-E	1	0	0.2	2-00-E	2	0	0.2
1-01-E	1	1	0.2	2-01-E	2	1	0.2
1-02-E	1	2	0.2	2-02-E	2	2	0.2
1-03-E	1	3	0.2	2-03-E	2	3	0.2
1-04-E	1	4	0.2	2-04-E	2	4	0.2
1-05-E	1	5	0.2	2-05-E	2	5	0.2
1-06-E	1	6	0.2	2-06-E	2	6	0.2

* Formulation F1, ** Formulation F2.

## Data Availability

Data available upon request.

## Supplementary Materials

The following supporting information can be downloaded at https://www.mdpi.com/article/10.3390/molecules29112695/s1, Table S1: DA oleoresin water miscibility; Table S2: Stability of Pickering emulsions at room temperature; Figure S1: HPLC Chromatogram wavelength 210 nm.
