# Supplementary material for: Pickering Emulsion of Oleoresin from Dipterocarpus alatus Roxb. ex G. Don and Its Antiproliferation in Colon (HCT116) and Liver (HepG2) Cancer Cells
Source: Molecules. 2024 Jun 6;29(11):2695. doi: 10.3390/molecules29112695 (PMC11174047; doi:10.3390/molecules29112695)

# Supplementary Material

# Pickering emulsion of oleoresin from *Dipterocarpus alatus* Roxb. ex G. Don and its antiproliferation in colon (HCT116) and liver (HepG2) cancer cells

**Piman Pocasap[1], Kawintra Tamprasit[2,3], Thanyathanya Rungsri[4], Karnchanok Kaimuangpak[2], Tarapong Srisonkram[3,5], Somporn Katekaew[6], Khanita Kamwilaisak[7], Ploenthip Puthongking[5], Natthida Weerapreeyakul[3,5]**

1 Department of Pharmacology, Faculty of Medicine, Khon Kaen University, Khon Kaen 40002, Thailand
2 Graduate School (in the program of Research and Development in Pharmaceuticals), Faculty of Pharmaceutical Sciences Khon Kaen University, Khon Kaen 40002, Thailand
3 Research Institute for Human High Performance and Health Promotion, Khon Kaen University, Khon Kaen 40002, Thailand
4 Faculty of Pharmaceutical Sciences (in the program of Doctor of Pharmacy), Khon Kaen University, Khon Kaen 40002, Thailand
5 Division of Pharmaceutical Chemistry, Faculty of Pharmaceutical Sciences, Khon Kaen University, Khon Kaen 40002, Thailand
6 Department of Biochemistry, Faculty of Sciences, Khon Kaen University, Khon Kaen, 40002, Thailand
7 Department of Chemical Engineering, Faculty of Engineering, Khon Kaen University, Khon Kaen, 40002, Thailand
* Correspondence: natthida@kku.ac.th; Tel.: +66-43202378

**Table S1:** DA oleoresin water miscibility.

| Sample | | Description |
|---|---|---|
| Oleoresin : water<br>Ratio 1 : 0<br>100 µl : 0 µl | 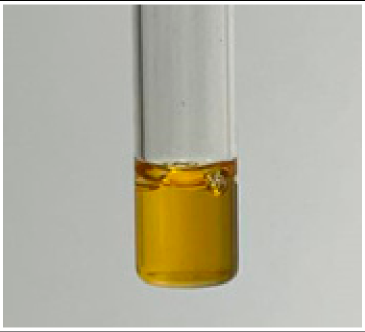 | Homogeneous liquid |
| Oleoresin : water<br>Ratio 1 : 0.1<br>100 µl : 10 µl | 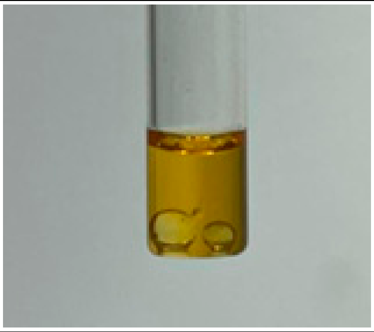 | Water immiscible<br><br>Oleoresin: Yellow<br>Water: Clear |
| Oleoresin : water<br>Ratio 1 : 0.3<br>100 µl : 30 µl | 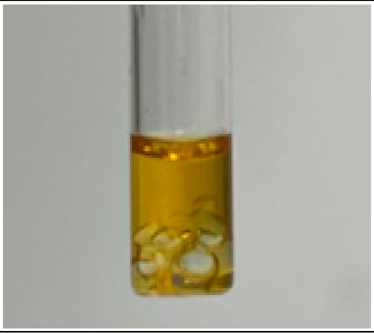 | Water immiscible |
| Oleoresin : water<br>Ratio 1 : 0.5<br>100 µl : 50 µl | 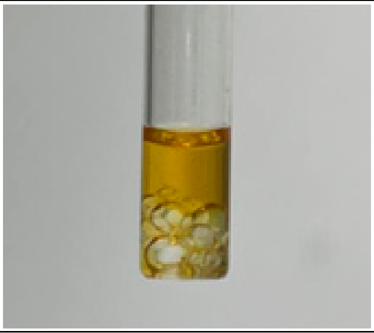 | Water immiscible |

| Sample | | Description |
|---|---|---|
| Oleoresin : water<br>Ratio 1 : 0.7<br>100 µl : 70 µl | 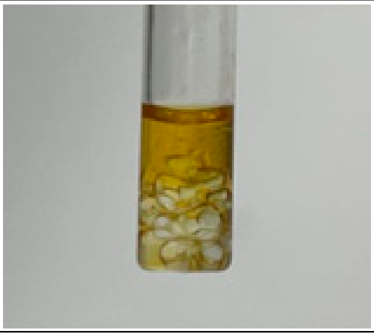 | Water immiscible |
| Oleoresin : water<br>Ratio 1 : 1<br>100 µl : 100 µl | 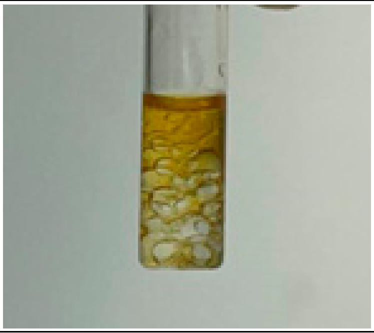 | Water immiscible |

**Table S2:** Stability of Pickering emulsions at room temperature.

| Formulations | Day 0 | Day 3 | Day 7 | Day 14 | Day 30 | Interpret |
|---|---|---|---|---|---|---|
| 1-00-D | 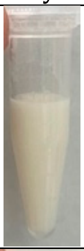 | 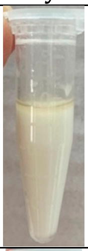 | 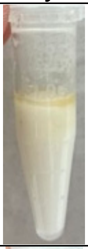 | 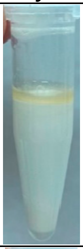 | 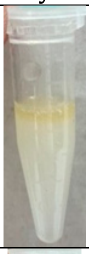 | Unstable |
| 1-01-D | 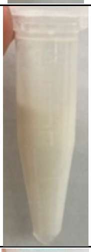 | 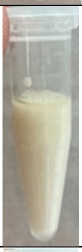 | 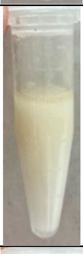 | 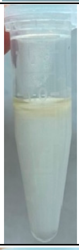 | 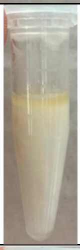 | Unstable |
| 1-02-D | 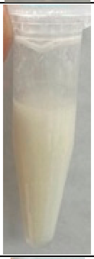 | 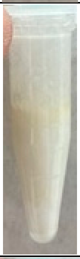 | 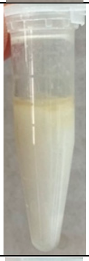 | 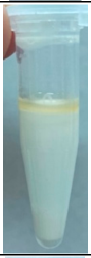 | 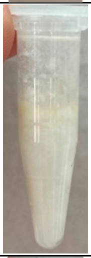 | Unstable |
| 1-03-D | 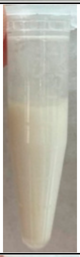 | 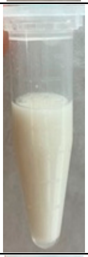 | 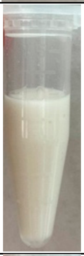 | 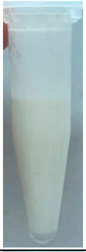 | 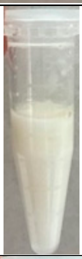 | Stable |
| 1-04-D | 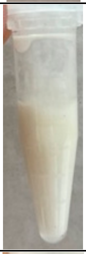 | 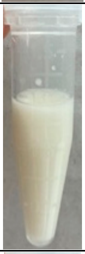 | 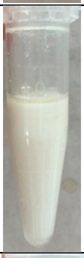 | 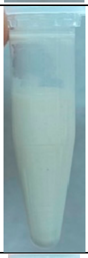 | 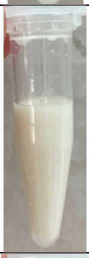 | Stable |
| 1-01-E | 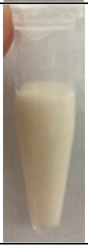 | 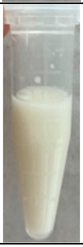 | 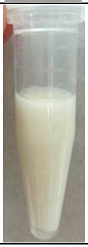 | 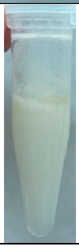 | 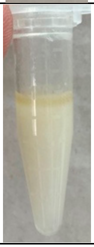 | Unstable |

| Formulations | Day 0 | Day 3 | Day 7 | Day 14 | Day 30 | Interpret |
|---|---|---|---|---|---|---|
| 1-02-E | 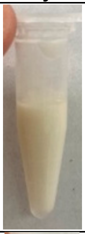 | 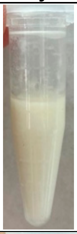 | 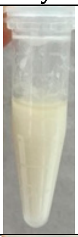 | 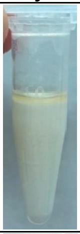 | 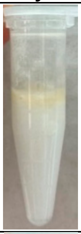 | Unstable |
| 1-03-E | 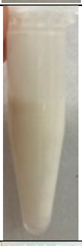 | 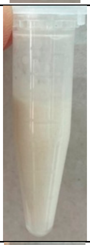 | 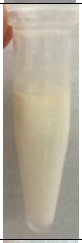 | 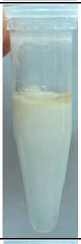 | 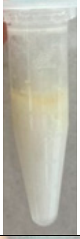 | Unstable |
| 2-01-D | 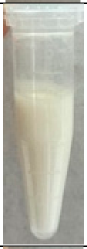 | 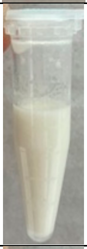 | 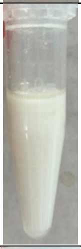 | 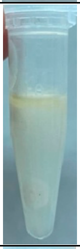 | 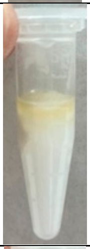 | Unstable |
| 2-02-D | 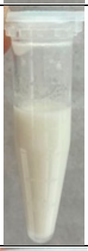 | 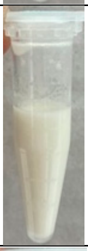 | 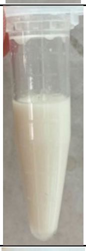 | 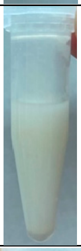 | 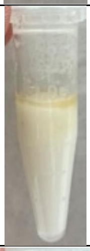 | Unstable |
| 2-03-D | 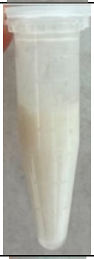 | 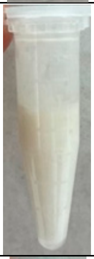 | 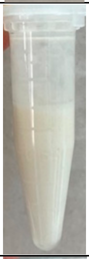 | 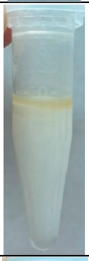 | 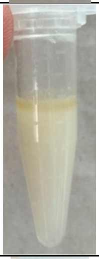 | Unstable |
| 2-04-D | 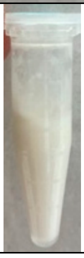 | 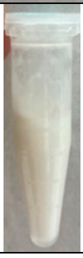 | 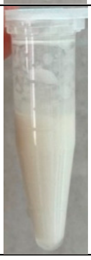 | 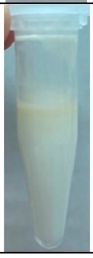 | 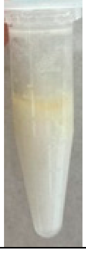 | Unstable |

| Formulations | Day 0 | Day 3 | Day 7 | Day 14 | Day 30 | Interpret |
|---|---|---|---|---|---|---|
| 2-01-E | 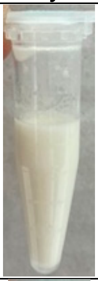 | 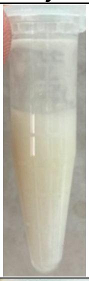 | 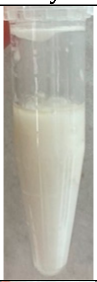 | 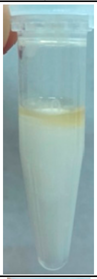 | 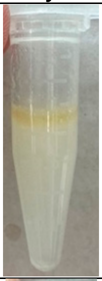 | Unstable |
| 2-02-E | 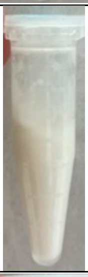 | 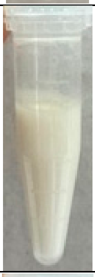 | 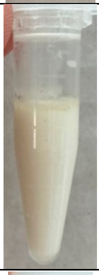 | 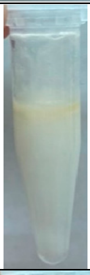 | 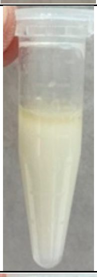 | Unstable |
| 2-03-E | 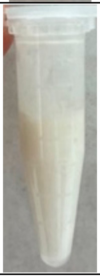 | 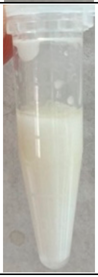 | 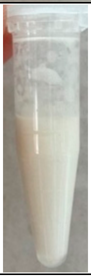 | 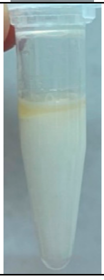 | 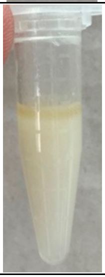 | Unstable |

**Figure S1:** HPLC Chromatogram wavelength 210 nm.

Dipterocarpol 100 µg/mL

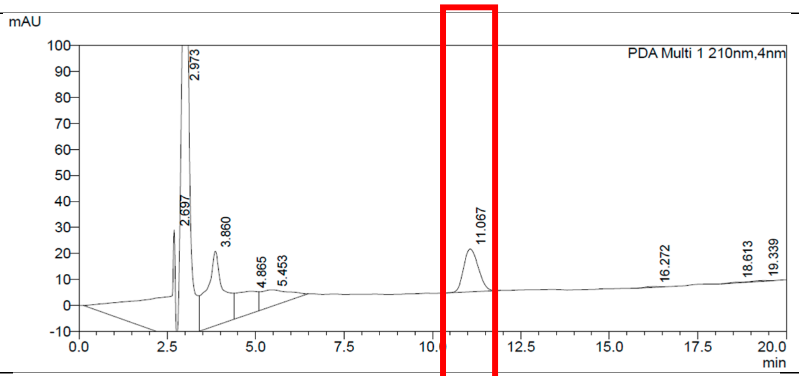


F1

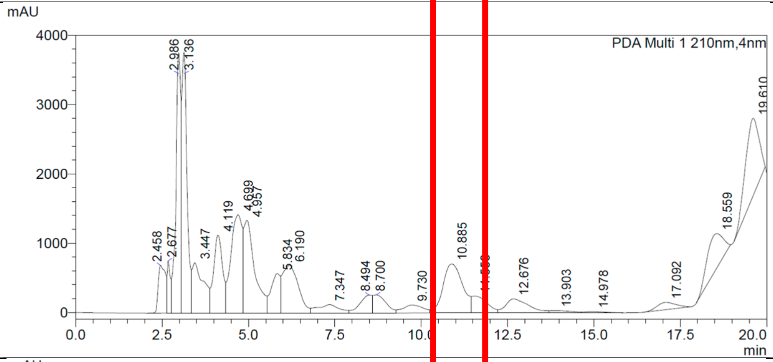


F2

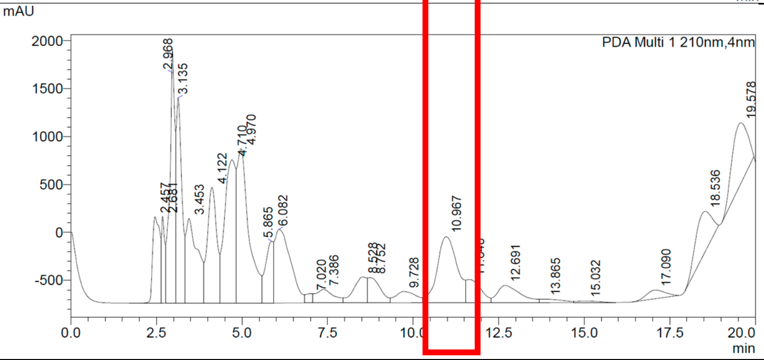

Supplement: Supplementary file 1 [file molecules-29-02695-s001.zip › molecules-3030465-supplementary.pdf]
